# Focused Cardiac Ultrasound Diagnosis of Cor Triatriatum Sinistrum in Pediatric Cardiac Arrest

**DOI:** 10.5811/westjem.2015.6.26093

**Published:** 2015-10-20

**Authors:** Thompson Kehrl, Callie T. Dagen, Brent A. Becker

**Affiliations:** WellSpan York Hospital, Department of Emergency Medicine, York, Pennsylvania

## Abstract

Cardiac arrest in the adolescent population secondary to congenital heart disease (CHD) is rare. Focused cardiac ultrasound (FoCUS) in the emergency department (ED) can yield important clinical information, aid in resuscitative efforts during cardiac arrest and is commonly integrated into the evaluation of patients with pulseless electrical activity (PEA). We report a case of pediatric cardiac arrest in which FoCUS was used to diagnose a critical CHD known as cor triatriatum sinistrum as the likely cause for PEA cardiac arrest and help direct ED resuscitation.

## INTRODUCTION

Congenital heart disease (CHD) is estimated to affect between four and thirteen of every 1,000 live births.^1^–^3^ Despite this relatively low incidence, CHD constitutes one of the leading causes of perinatal and infant death.^4^ The most common defect is ventricular septal defect (VSD), followed in frequency by atrial septal defect (ASD) and patent ductus arteriosus.^3^ Critical CHD, defined as lesions requiring operative intervention within the first year of life, occur in approximately 25% of those with CHD.^5^ Outcomes for patients with CHD are time-dependent, with early diagnosis and intervention improving morbidity and mortality.^6^

Cardiac arrest is rare in the pediatric population with an estimated incidence of 0.5 to 20 per 100,000 person years. While the majority of cardiac arrest in infants and young children are attributed to CHD, rates are lower in adolescents. For patients between 14 and 24 years of age, CHD has been implicated in 23% of cases of cardiac arrest while arrhythmias also constitute 23% of cases. Less common etiologies include dilated cardiomyopathy, long QT syndrome, myocarditis and hypertrophic cardiomyopathy.^7^–^8^

Emergency physicians and other non-cardiologists use focused cardiac ultrasound (FoCUS) to expedite diagnosis and direct real-time patient management. FoCUS augments clinical decision-making in the care of critically ill patients. It is particularly helpful in differentiating among the potential causes of shock and cardiac arrest and has been shown to change management in a majority of patients with pulseless electrical activity (PEA) cardiac arrest.^9^–^10^

In this article, we present a case of an adolescent out-of-hospital cardiac arrest due to a very rare form of CHD, known as cor triatriatrum sinistrum (CTS), in which FoCUS was instrumental in the diagnosis and management of the patient.

## CASE REPORT

A 15-year old female with unknown past medical history presented to the emergency department (ED) after collapsing while walking. She was found to be acutely dyspneic by family and soon became pulseless. Bystander cardiopulmonary resuscitation (CPR) was initiated and emergency medical services was summoned. Upon arrival to the scene paramedics noted the patient to be in PEA. CPR was continued, bag-valve-mask ventilation was initiated, intravenous (IV) access was established and IV epinephrine was administered. Endotracheal intubation was attempted twice en route without success and was complicated by copious vomiting. Upon arrival to the ED, CPR was continued and the trachea was intubated. A venous blood gas showed a pH of 6.89, pCO_2_ 43mmHg, bicarbonate 8mmol/L, and a base excess of −24. After four minutes of CPR, an increase in end-tidal CO_2_ was noted and subsequent pulse check confirmed return of spontaneous circulation (ROSC).

**Figure 1:**
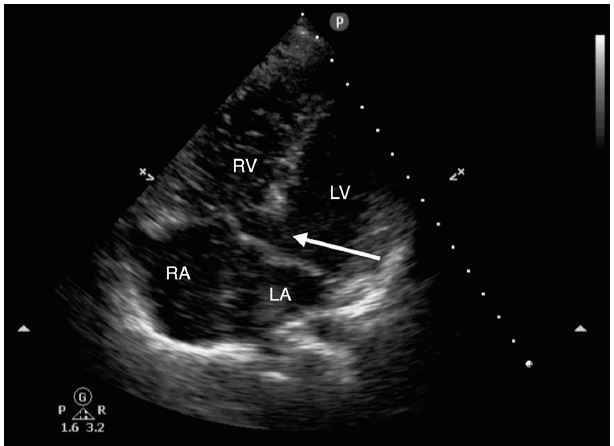
Apical four chamber view, slightly off axis, with a large ventricular septal defect (arrow). *RV*, right ventricle; *LV*, left ventricle; *RA*, right atrium; *LA*, left atrium

**Figure 2:**
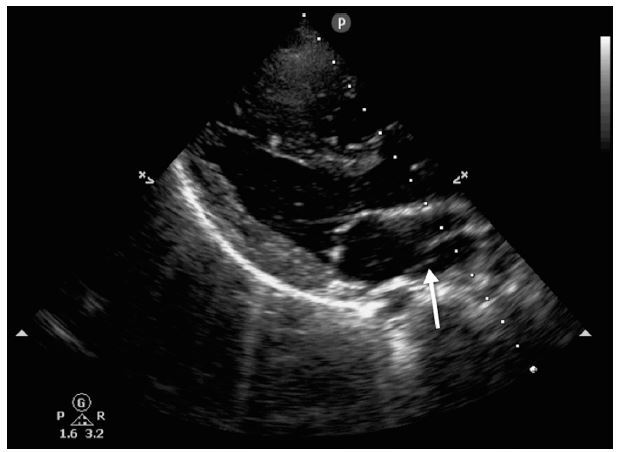
Parasternal long axis view showing double left atrium (arrow).

The treating emergency physicians then performed a FoCUS using a phased-array probe (Phillips, Andover, MA). Images obtained in the parasternal and apical windows demonstrated a globally hypokinetic left ventricle with significant spontaneous echo contrast in all four chambers of the heart. The apical view demonstrated spontaneous echo contrast passing freely between the left and right ventricles through a large VSD (Figure 1). On the parasternal long axis view, the VSD was confirmed and a septation in the left atrium was noted (Figure 2, Video). FoCUS allowed for the emergency physicians to recognize the patient’s congenital heart defect as the probable cause of the cardiac arrest. Approximately two minutes after ROSC, the patient’s end-tidal CO_2_ dropped precipitously and pulses were lost despite ongoing electrical activity on the monitor. ROSC was regained briefly after three additional rounds of CPR and IV epinephrine, but was subsequently lost again. Cardiothoracic surgery was consulted emergently as it was felt that initiation of extracorporeal membrane oxygenation (ECMO) would potentially stabilize the patient and allow bridge to definitive operative repair. Attempts were made at both percutaneous cannulation and cut-down of the femoral vessels to allow for placement of ECMO cannulae but were ultimately unsuccessful and resuscitative efforts were halted. Post-mortem autopsy was requested by the family and revealed a large VSD, CTS and evidence of biventricular heart failure.

## DISCUSSION

CTS is a relatively rare congenital cardiac anomaly, found only in 0.1% of all children with CHD. First described in 1868, CTS is characterized by an abnormal septation of the left atrium by a fibromuscular membrane that divides the atrium into a proximal and distal chamber.^11^–^12^ These chambers communicate by one or more fenestrations of the membrane and the amount of flow between the two chambers determines the severity of the lesion. CTS is often associated with other defects, most commonly a patent foramen ovale or ASD. The mortality of untreated CTS is significant. Without surgical removal of the extraneous atrial membrane, 75% of patients die in infancy or childhood; however, survival rates after surgical correction are excellent. While most cases of CTS are diagnosed in infancy, there are cases of the diagnosis being delayed by decades.^13^

CHD should be considered in all adolescent patients with sudden cardiac arrest, even if not diagnosed previously. Massin et al. demonstrated that approximately 10% of infants with cyanotic lesions are not diagnosed before discharge from the hospital. Furthermore, of those children with acyanotic lesions mandating surgical repair, 35.1% presented with hemodynamic instability requiring emergent intervention at an age beyond that recommended for elective repair.^14^

FoCUS is an important tool for the acute care clinician caring for the critically ill pediatric patient with or without known CHD. While there is a paucity of literature specifically examining the utility of FoCUS in pediatric patients, research in adults suggests it can effectively identify potentially reversible causes of PEA, such as cardiac tamponade due to pericardial effusion or structural heart defects.^15^–^16^ There are multiple proposed algorithms for integration of FoCUS into the evaluation of PEA with each one highlighting the recognition of potentially reversible causes.^17^–^19^ In this case, FoCUS facilitated the identification of CHD as the likely etiology for the patient’s arrest, prompting emergent consultation with cardiothoracic surgery and attempts at ECMO. In summary, we present a case of an adolescent cardiac arrest due to a congenital heart defect that was discovered by FoCUS. This case highlights the need to consider CHD in the differential diagnosis of adolescent cardiac arrest and the utility of FoCUS in the management of critically ill pediatric patients.

## Figures and Tables

**Video:** Parasternal long axis view with large ventricular septal defect, double left atrium, and spontaneous echo contrast freely moving between the right and left ventricles.
